# Acute Isolated Thenar Compartment Syndrome in a Patient with Evans Syndrome: A Case Report

**DOI:** 10.5811/cpcem.2022.8.57193

**Published:** 2022-11-04

**Authors:** Elisabeth Jiang, Kevin H. Kim, Alan Babigian

**Affiliations:** * University of Connecticut School of Medicine, Emergency Medicine Residency, Farmington, Connecticut; † University of Connecticut School of Medicine, Hand Surgery Fellowship, Farmington, Connecticut; ‡ Hartford Hospital, Department of Surgery, Hartford, Connecticut

**Keywords:** case report, atraumatic compartment syndrome, Evans syndrome, hand compartment syndrome

## Abstract

**Introduction:**

Acute compartment syndrome of the hand is a rare medical emergency, most often associated with trauma or fracture.

**Case Report:**

Here, we describe a rare case of isolated thenar compartment syndrome of the hand in the absence of major trauma as the presenting symptom of pancytopenia due to Evans syndrome, an uncommon autoimmune hematologic disorder.

**Conclusion:**

In cases of atraumatic compartment syndrome, it is critical to evaluate for underlying coagulopathy in patients presenting to the emergency department.

## INTRODUCTION

Hand compartment syndrome is a rare but critical condition that emergency physicians must recognize. As with any acute compartment syndrome (ACS), it is a true surgical emergency. Missed or delayed diagnosis is likely to result in devastating functional loss to the patient. While most cases of compartment syndrome are associated with bone fractures and major trauma, ACS can also occur in the absence of fracture, and atraumatic ACS is an easily missed diagnosis if the emergency physician does not maintain a high clinical index of suspicion. It is crucial for physicians to understand the anatomy and pathophysiology of hand compartment syndrome to ensure early diagnosis and prompt surgical intervention.

The hand comprises 11 compartments separated by inflexible fascial membranes; an increase in pressure within any of these compartments can result in decreased perfusion, tissue death, and ultimately loss of function of the hand. These compartments include four dorsal interossei compartments, three volar interossei compartments, the thenar compartment, the hypothenar compartment, the adductor compartment, and the midpalm compartment^1^ (Figure). The blood supply to the hand is provided by branches of the deep and superficial palmar arches, which are fed by both the radial and ulnar arteries.^1^

Here, we describe a rare case of isolated thenar compartment syndrome of the hand in the absence of major trauma as the presenting symptom of pancytopenia in a previously healthy 17-year-old high school athlete. This is the first reported case in the literature to describe ACS as the presenting clinical manifestation of Evans syndrome.

## CASE REPORT

A 17-year-old, previously healthy right-hand dominant male presented to the emergency department (ED) with pain, swelling, and numbness in his left hand. Three days prior to presentation, the patient had injured his left hand while playing football. He could not recall a specific incident leading to his hand injury such as a fall or tackle; rather he noticed pain and swelling in his hand following a football game, which continued to worsen in the following days. He was seen at an outside hospital where imaging was negative for fracture or dislocation. However, he continued to experience worsening pain, paresthesia, and swelling of his thumb and hand predominately located in the thenar compartment.

On physical exam, the patient had exquisite tenderness over the left thenar eminence and thumb with significant swelling in the thenar compartment, without any overlying erythema or open wounds. The patient had severely limited range of motion in the wrist as well as the metacarpophalangeal and interphalangeal joints of the thumb secondary to severe pain. He was unable to oppose his thumb and any passive motion of the thumb caused significant pain. He also reported decreased sensation on the radial aspect of his left thumb. Sensation, capillary refill, strength, and range of motion were normal in digits two through five.

Radiographs were repeated in the ED, again revealing no bony injuries. Despite reassuring imaging, the patient’s clinical presentation, with tense swelling of the left thenar compartment, pain, decreased range of motion, and sensory deficit raised concern for compartment syndrome. Compartment pressures of the thenar compartment of the left hand were obtained using a Stryker needle with readings of 72 and 73 millimeters of mercury (mm Hg), with a diastolic blood pressure of 80 mm Hg. A diagnosis of compartment syndrome was made, and the patient was taken to the operating room for emergent fasciotomy.

Prior to being transported to the operating room (OR), the patient’s bloodwork resulted, revealing pancytopenia with profound neutropenia, thrombocytopenia, anemia, and leukopenia (Table). He denied any known history of bleeding disorder, cancer, recent viral illness, or easy bruising or bleeding. In the OR, emergent fasciotomy of the thenar compartment of the left hand was performed. The surgical team decided to release only the thenar compartment due to bleeding risk and because the patient did not show any signs of compartment syndrome in the other hand compartments. The incision was primarily closed with loosely approximated sutures; we did not use negative pressure device or leave the wound open due to bleeding and infection risk given the patient’s pancytopenic state. Two units of platelets were transfused intraoperatively.

**CPC-EM Capsule**

What do we already know about this clinical entity?

*Compartment syndrome is true medical emergency and early recognition by the emergency physician is critical to prevent devastating functional loss*.

What makes this presentation of disease reportable?

*Compartment syndrome of the hand is rare and typically associated with fractures, however in this case it occurred in a young patient in the absence of broken bones*.

What is the major learning point?

*Emergency physicians must maintain a high clinical suspicion for compartment syndrome in order to ensure timely diagnosis and treatment with surgical fasciotomy*.

How might this improve emergency medicine practice?

*Compartment syndrome should always be a consideration in cases of limb swelling, and further medical workup is needed in cases of atypical compartment syndrome*.

The patient was subsequently admitted to the hospital for hematology/oncology workup of pancytopenia. Peripheral blood smear showed no abnormal white blood cells, reduced platelets, few giant platelets, and normal red blood cells without spherocytes. Bone marrow aspirate revealed blasts within normal range and slightly elevated monocytes. Viral workup was negative for human immunodeficiency virus, hepatitis A, B, and C, cytomegalovirus, Epstein-Barr virus, and parvovirus B19. Folate and vitamin B12 levels were within normal limits. Partial thromboplastin time was within normal limits and double-stranded DNA was negative, both reassuring for absence of lupus. Empiric treatment with intravenous immunoglobulin and dexamethasone was initiated for concern of underlying autoimmune process. A bone marrow biopsy was performed, which revealed hypercellular marrow with megakaryocytes and granulocytic left shift.

Ultimately, a diagnosis of Evans syndrome was made given the clinical presentation of concomitant autoimmune hemolytic anemia (AIHA) and immune thrombocytopenia (ITP). In this case the patient also presented with autoimmune neutropenia, a less common manifestation of Evans syndrome.^2^

The patient was discharged from the hospital on postoperative day five, with discharge labs revealing absolute neutrophil count of 810 units per liter (uL), platelets of 115,000/uL, hemoglobin 10.5 grams per deciliter, hematocrit 31.1%, and white blood cell count of 3,000/uL. He was seen for follow-up in the senior author’s office on postoperative day 14. At that time his incision was well healed; however, there was some residual stiffness of metacarpophalangeal and interphalangeal joints, with continued numbness of the radial aspect of the thumb. The patient was discharged on a four-day course of dexamethasone. Upon follow-up with the hematology/oncology service, blood work did reveal persistent pancytopenia, and he was scheduled to begin rituximab infusions to manage his autoimmune pancytopenia.

In this case, the diagnosis of isolated thenar compartment syndrome was likely a blessing in disguise; the patient was instructed to refrain from contact sports due to his high risk of life-threatening bleeding, and he was given appropriate follow-up for his autoimmune pancytopenia.

## DISCUSSION

We describe a case of acute thenar compartment syndrome in a previously healthy 17-year-old male who was subsequently diagnosed with Evans syndrome, a rare autoimmune cytopenia syndrome.

Acute compartment syndrome results from increased pressure in any of the body compartments that are contained within an inflexible fascial membrane, causing impairment of local circulation. The incidence of ACS is estimated to be 7.3 per 100,000 in males and 0.7 per 100,000 in females.^3^ The leg and forearm are the most frequently affected sites; however, it can also involve the arm, hand, foot, and buttock. Incidence is highest in young men and most often occurs after traumatic injuries; 75% of cases of compartment syndrome are associated with bone fractures.^3^

Compartment syndrome may occur in the setting of traumatic injuries not involving fractures. These include burns, crush injuries, constrictive bandages, splints or casts, penetrating trauma, high pressure injection injuries, vascular injuries, and animal bites and stings. There are also cases of non-traumatic compartment syndrome, which may be seen in the setting of bleeding disorders, anticoagulation use, nephrotic syndrome, toxic envenomation, massive fluid resuscitation, revascularization procedures, and muscle infection.^4^ As demonstrated by Hope et al, cases of compartment syndrome not associated with acute fracture often go undiagnosed and there is a risk of delayed treatment with more devastating sequela and functional loss.^5^ Specifically, Hope et al reported that patients without the presence of fracture were more likely to have muscle necrosis at the time of fasciotomy than patients with ACS in the setting of acute fracture.

Generally, ACS is a clinical diagnosis, and it is often diagnosed based on history and physical examination. However, intracompartmental pressure is sometimes measured to confirm the diagnosis in uncertain situations as these measurements offer the only objective means of diagnosis and may be the only tool to make a diagnosis of ACS in the case of obtunded patients or unusual presentations.^1^ Diagnosis of ACS is often made by using delta pressure, the difference between diastolic blood pressure and compartment pressure, with a delta less than 30 mm Hg concerning for ACS.^6^ Other recommendations for emergent fasciotomy are based on an absolute compartment pressures greater than 30 or greater than 45 mm Hg.^7^

When faced with a swollen hand, the decision to obtain compartment pressures is based on several factors including a detailed history, physical exam, and overall clinical suspicion for ACS. Findings historically associated with ACS (pain, paresthesia, pallor, paralysis, and pulselessness) are often late signs of vascular compromise; so the decision to proceed with compartment pressure measurement and ultimately fasciotomy must often be made in the absence of these clues. Due to the risk of permanent functional loss of the hands, any clinical suspicion for hand compartment syndrome should potentially warrant compartment pressure measurement and surgical consultation. There has been some investigation into the role of laboratory markers as an aid to diagnose compartment syndrome in the ED setting. In a 2020 retrospective study by Weingart et al, several markers were identified that showed correlation with compartment syndrome including creatinine kinase, bicarbonate, ionized calcium, lactic acid, creatinine, blood urea nitrogen, and potassium. This study demonstrated a sensitivity of only 2.9%, indicating that serum markers are not useful in helping to clinically rule out a diagnosis of ACS, limiting their utility in the ED setting.^14^

Evans syndrome, first described in 1951, is defined as the concomitant or sequential occurrence of immune thrombocytopenia and autoimmune hemolytic anemia.^8^ It is a rare syndrome, accounting for only 7% of AIHA cases and 2% of ITP cases.^2^ Evans syndrome may exist as a primary pathology; however, in up to 50% of cases it is secondary to another underlying pathology such as hematologic malignancy, systemic lupus erythematosus, viral infection, or primary immunodeficiency. The first line therapy for treatment of ES is corticosteroids, while IVIG use is typically reserved for patients with platelet count less than 20,000. Rituximab and mycophenolate mofetil have also been used in treatment of ES, and there are case reports citing hematopoietic stem cell transplantation and splenectomy.^9^

## CONCLUSION

This report illustrates a rare case of thenar compartment syndrome in a patient with a rare hematologic condition. As demonstrated in previous studies, cases of compartment syndrome not associated with high energy injuries often go undiagnosed, which often results in delayed treatment with more devastating sequela and functional loss.^5^ Emergency physicians should have a high clinical suspicion for acute compartment syndrome, and if there is no obvious explanation for the patient’s symptoms, a prompt hematological workup is warranted to investigate other predisposing factors for bleeding such as thrombocytopenia, hematologic malignancy, or other coagulopathy.

## Figures and Tables

**Figure:**
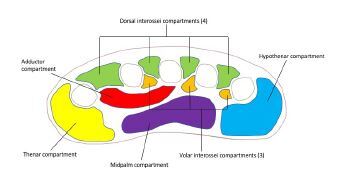
Compartments of the hand.

**Table:** Emergency department laboratory results.

	Value	Reference range
White blood cell count	2,200 per microliter	4,000 – 11,000 per microliter
Hemoglobin	10.7 grams/deciliter	13.0 – 17.7 grams/deciliter
Hematocrit	31.7%	39.0 – 54.0%
Platelets	Less than 2,000 per microliter	150,000 – 450,000 per microliter
Absolute neutrophil count	100 per microliter	2,000 – 7,500 per microliter
